# Supplementary material for: Assessment of the Spatial Invasion Risk of Intentionally Introduced Alien Plant Species (IIAPS) under Environmental Change in South Korea
Source: Biology (Basel). 2021 Nov 12;10(11):1169. doi: 10.3390/biology10111169 (PMC8614709; doi:10.3390/biology10111169)
Supplement: Supplementary file 1 [file biology-10-01169-s001.zip › Table S1.pdf]

Table S1. Spearman's correlation for bioclimatic variable selection

Variable	**Bio1**	Bio2	**Bio3**	**Bio4**	Bio5	Bio6	Bio7	**Bio8**	Bio9	Bio 10	Bio 11	**Bio 12**	**Bio 13**	**Bio 14**	Bio 15	Bio16	Bio 17	Bio 18	Bio 19	**d-water**	**d-road**
Bio2	-0.27																				
**Bio3**	0.02	0.83																			
**Bio4**	-0.60	0.49	-0.04																		
Bio5	0.80	0.21	0.29	-0.14																	
Bio6	0.92	-0.48	-0.08	-0.81	0.57																
Bio7	-0.55	0.75	0.29	0.93	-0.02	-0.79															
Bio8	0.90	-0.05	0.08	-0.28	0.93	0.72	-0.24														
Bio9	0.94	-0.35	0.05	-0.79	0.64	0.98	-0.71	0.76													
Bio10	0.85	-0.04	0.01	-0.16	0.93	0.64	-0.13	0.97	0.68												
Bio11	0.96	-0.35	0.04	-0.78	0.65	0.98	-0.71	0.78	0.99	0.70											
**Bio12**	-0.06	-0.26	-0.19	-0.19	-0.21	0.03	-0.22	-0.15	0.02	-0.15	0.00										
**Bio13**	-0.54	0.17	-0.17	0.64	-0.34	-0.60	0.54	-0.38	-0.59	-0.30	-0.61	0.46									
**Bio14**	0.07	-0.34	-0.21	-0.33	-0.14	0.21	-0.38	-0.01	0.15	-0.05	0.15	0.17	-0.27								
Bio15	-0.53	0.39	-0.02	0.84	-0.18	-0.68	0.75	-0.27	-0.66	-0.19	-0.66	-0.03	0.80	-0.58							
Bio16	-0.36	-0.06	-0.25	0.32	-0.33	-0.35	0.23	-0.33	-0.35	-0.26	-0.38	0.73	0.87	-0.26	0.55						
Bio17	0.24	-0.43	-0.15	-0.62	-0.10	0.41	-0.62	0.04	0.37	-0.03	0.37	0.26	-0.45	0.91	-0.81	-0.29					
Bio18	-0.40	0.09	-0.10	0.39	-0.29	-0.41	0.33	-0.32	-0.40	-0.27	-0.42	0.66	0.91	-0.26	0.62	0.95	-0.34				
Bio19	0.23	-0.43	-0.16	-0.61	-0.09	0.40	-0.61	0.05	0.36	-0.02	0.36	0.27	-0.44	0.90	-0.82	-0.28	0.99	-0.33			
**d-water**	-0.28	0.05	0.09	-0.04	-0.32	-0.19	-0.01	-0.33	-0.19	-0.36	-0.20	0.06	-0.01	0.08	-0.06	0.01	0.12	0.01	0.12		
**d-road**	-0.31	-0.08	-0.07	-0.04	-0.43	-0.21	-0.05	-0.41	-0.23	-0.41	-0.24	0.21	0.12	0.15	-0.04	0.17	0.16	0.14	0.15	0.16	
**SSP1**	-0.09	0.00	0.03	-0.02	-0.14	-0.15	-0.02	-0.12	-0.06	-0.13	-0.16	0.12	0.22	-0.11	0.01	0.13	0.01	0.03	0.02	0.15	0.01
