# Supplementary material for: Assessment of the Spatial Invasion Risk of Intentionally Introduced Alien Plant Species (IIAPS) under Environmental Change in South Korea
Source: Biology (Basel). 2021 Nov 12;10(11):1169. doi: 10.3390/biology10111169 (PMC8614709; doi:10.3390/biology10111169)
Supplement: Supplementary file 1 [file biology-10-01169-s001.zip › Table S2.pdf]

Table S2. List of bioclimatic and environmental variables used in this study.

Code	Description	Unit	Source
Bio01	Annual mean temperature	Degrees Celsius	KMA
Bio03	Isothermality (BIO2/BIO7) (* 100)	Percentage	KMA
Bio04	Temperature seasonality	Percentage	KMA
Bio12	Annual precipitation	Millimeters	KMA
Bio13	Precipitation in the wettest month	Millimeters	KMA
Bio14	Precipitation in the driest month	Millimeters	KMA
d-road	Distance from roads	Meters	This study
d-water	Distance from water	Meters	This study
SSP1	Land cover	-	Song et al. (2018)

KMA, Korea Meteorological Administration.
