# Supplementary material for: Assessment of the Spatial Invasion Risk of Intentionally Introduced Alien Plant Species (IIAPS) under Environmental Change in South Korea
Source: Biology (Basel). 2021 Nov 12;10(11):1169. doi: 10.3390/biology10111169 (PMC8614709; doi:10.3390/biology10111169)
Supplement: Supplementary file 1 [file biology-10-01169-s001.zip › Figure S1_Species presence and Spatial distribution of IIPS.pdf]

Figure S1a-j. Spatial distribution of intentionally introduced alien plant species in South Korea. S1a, *Amorpha fruticosa*; S1b, *Coreopsis lanceolata*; S1c, *Dactylis glomerata*; S2d, *Eragrostis curvula*; S1e, *Ageratina altissima* ; S1f, *Festuca arundinacea*; S1g, *Helianthus tuberosus*; S1h, *Lolium perenne*; S1i, *Medicago sativa*; S1j, *Poa pratensis.*

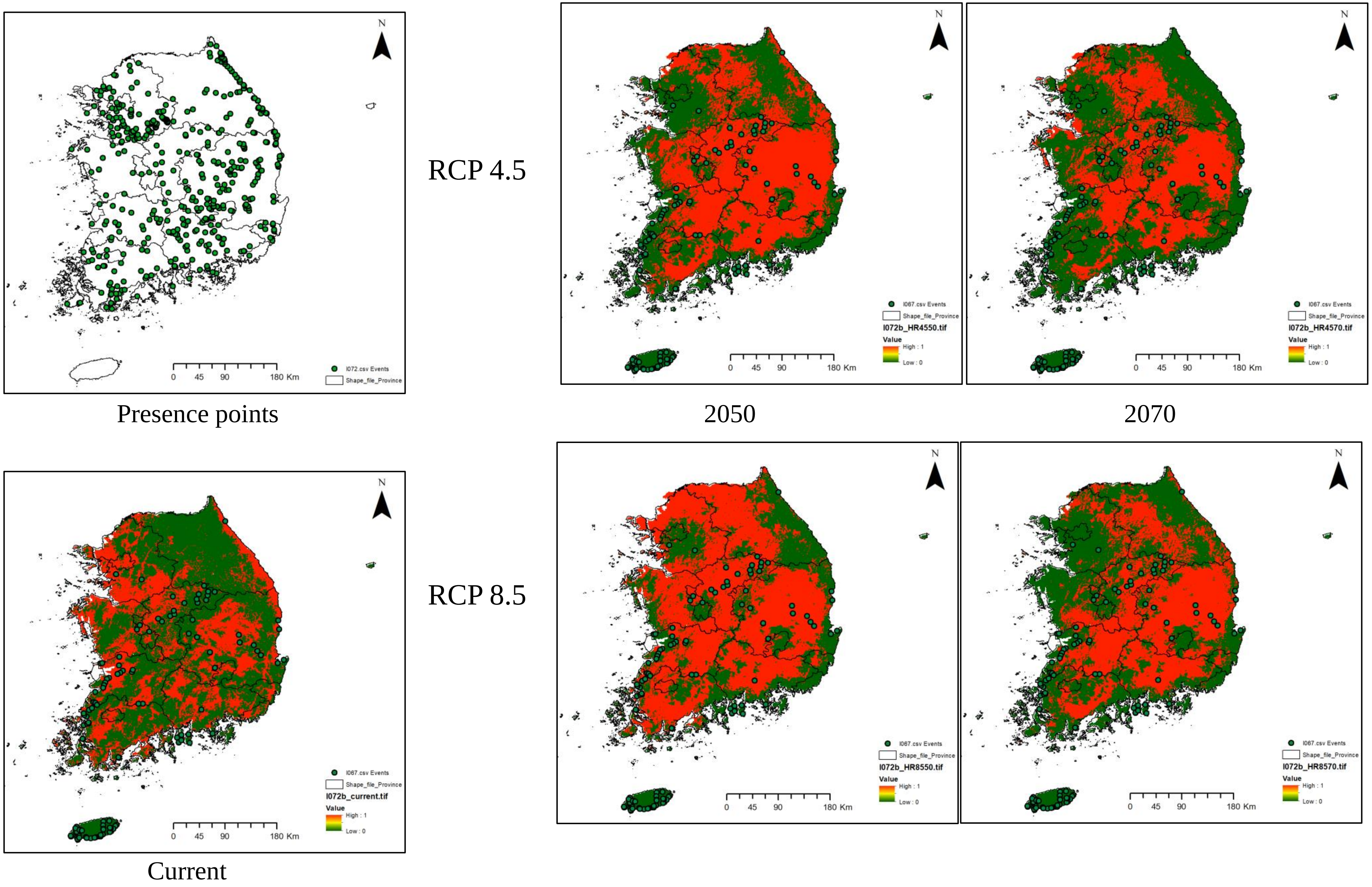

Figure S1a. Spatial distribution of *Amorpha fruticosa* (I072) under RCP 4.5 and RCP 8.5.

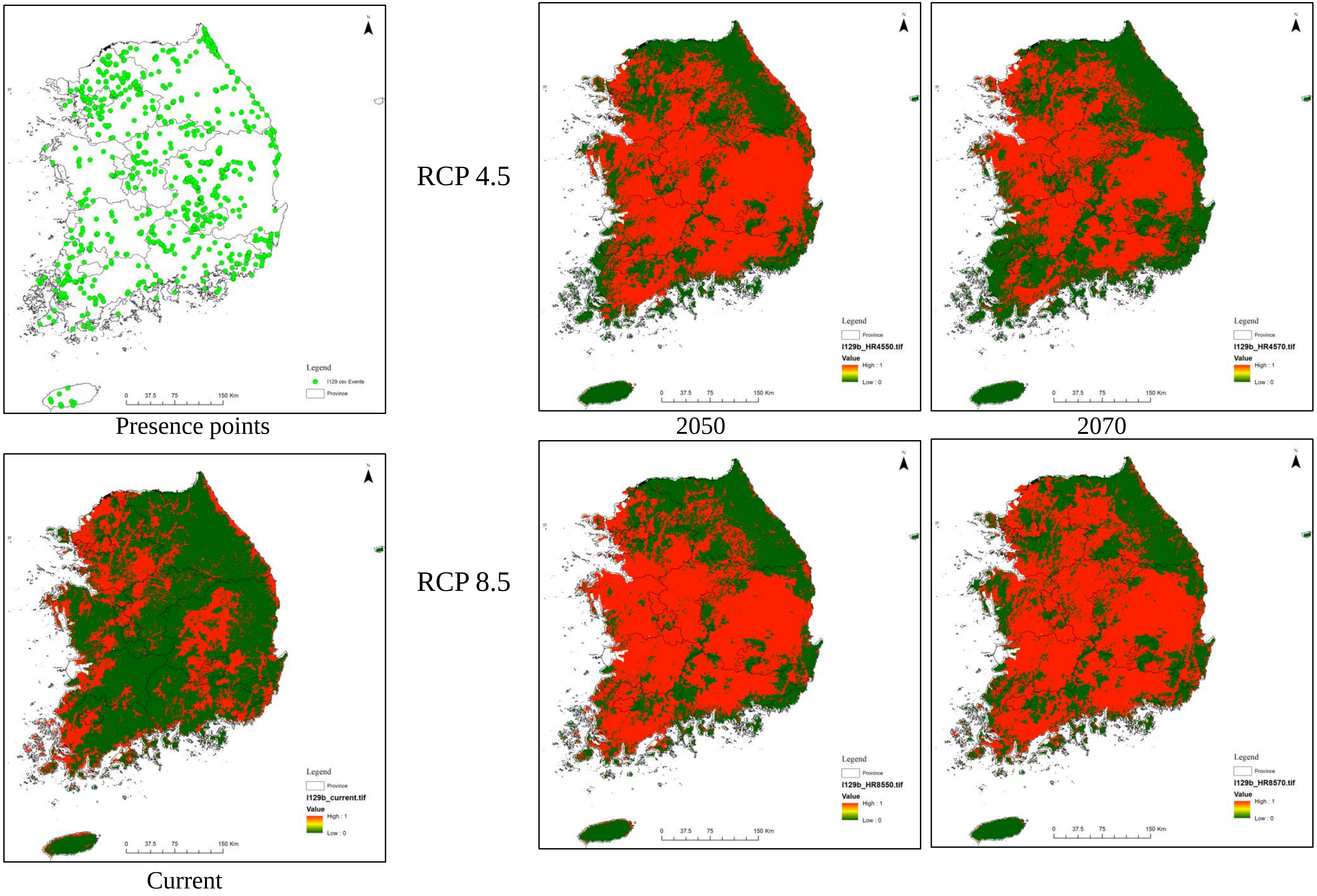

Figure S1b. Spatial distribution of *Coreopsis lanceolate* (I129) under RCP 4.5 and RCP 8.5.

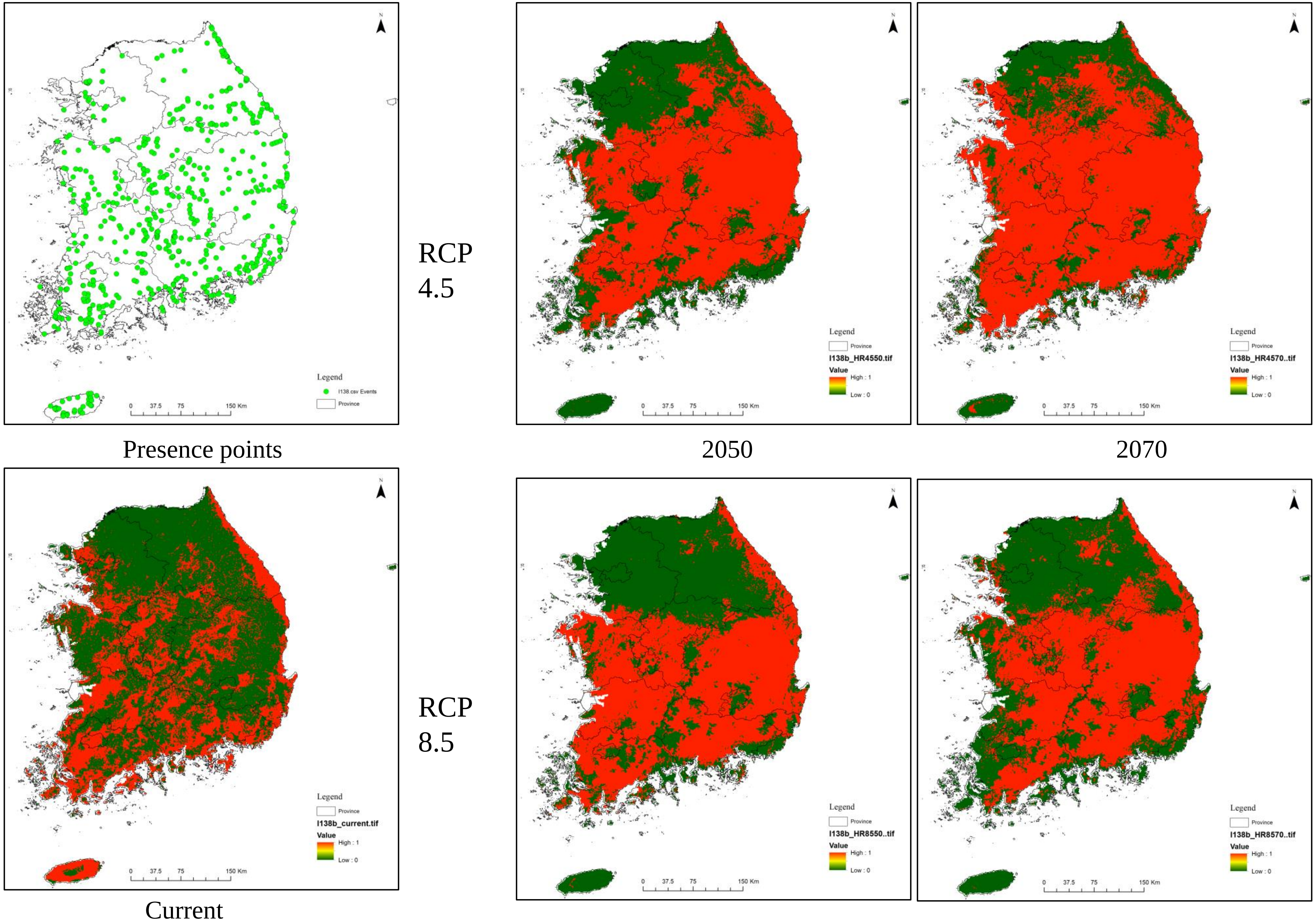

Figure S1c. Spatial distribution of *Dactylis glomerate* (I138) under RCP 4.5 and RCP 8.5.

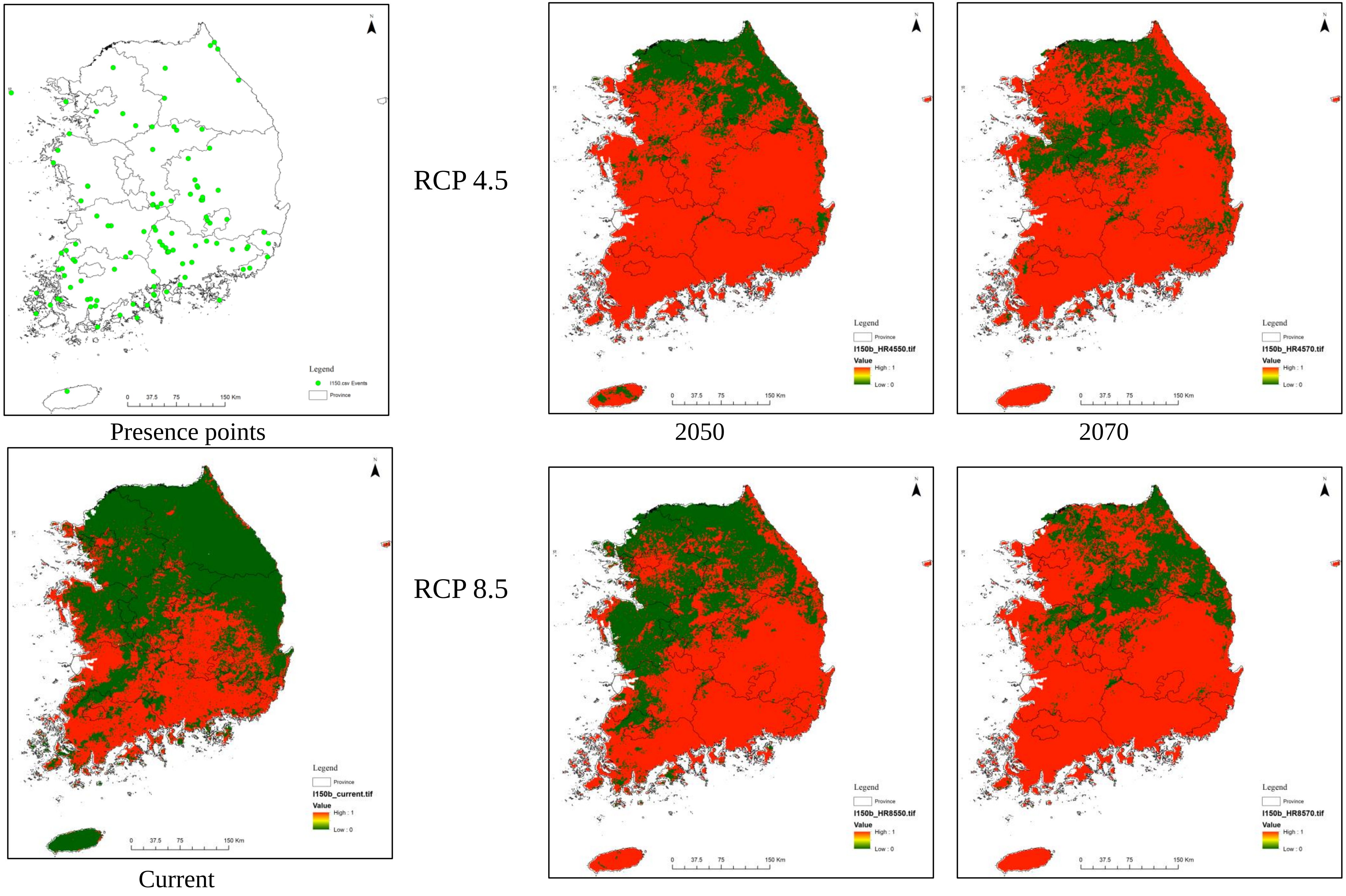

Figure S1d. Spatial distribution of *Eragrostis curvula* (I150) under RCP 4.5 and RCP 8.5.

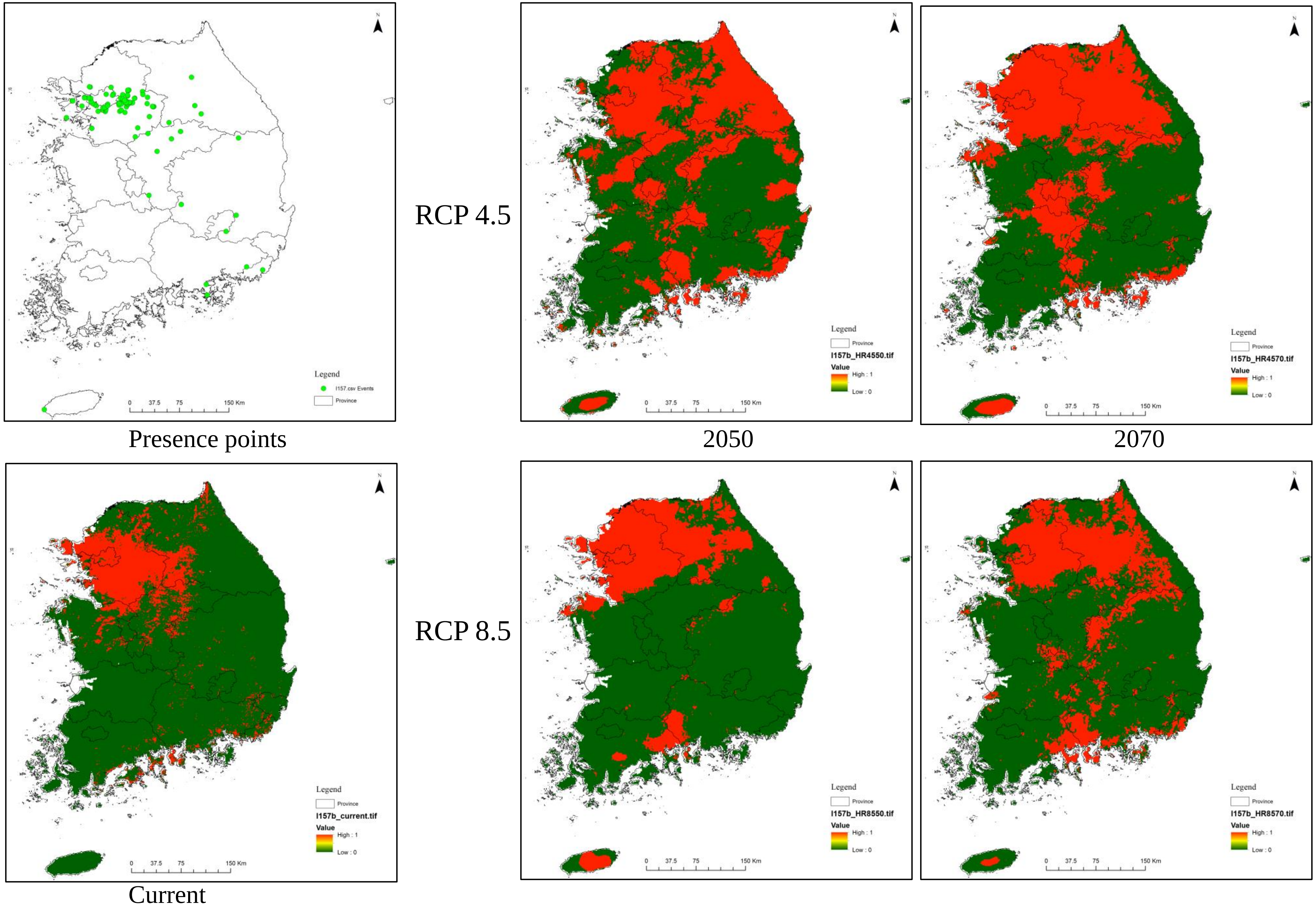

Figure S1e. Spatial distribution of *Ageratina altissima* (I157) under RCP 4.5 and RCP 8.5.

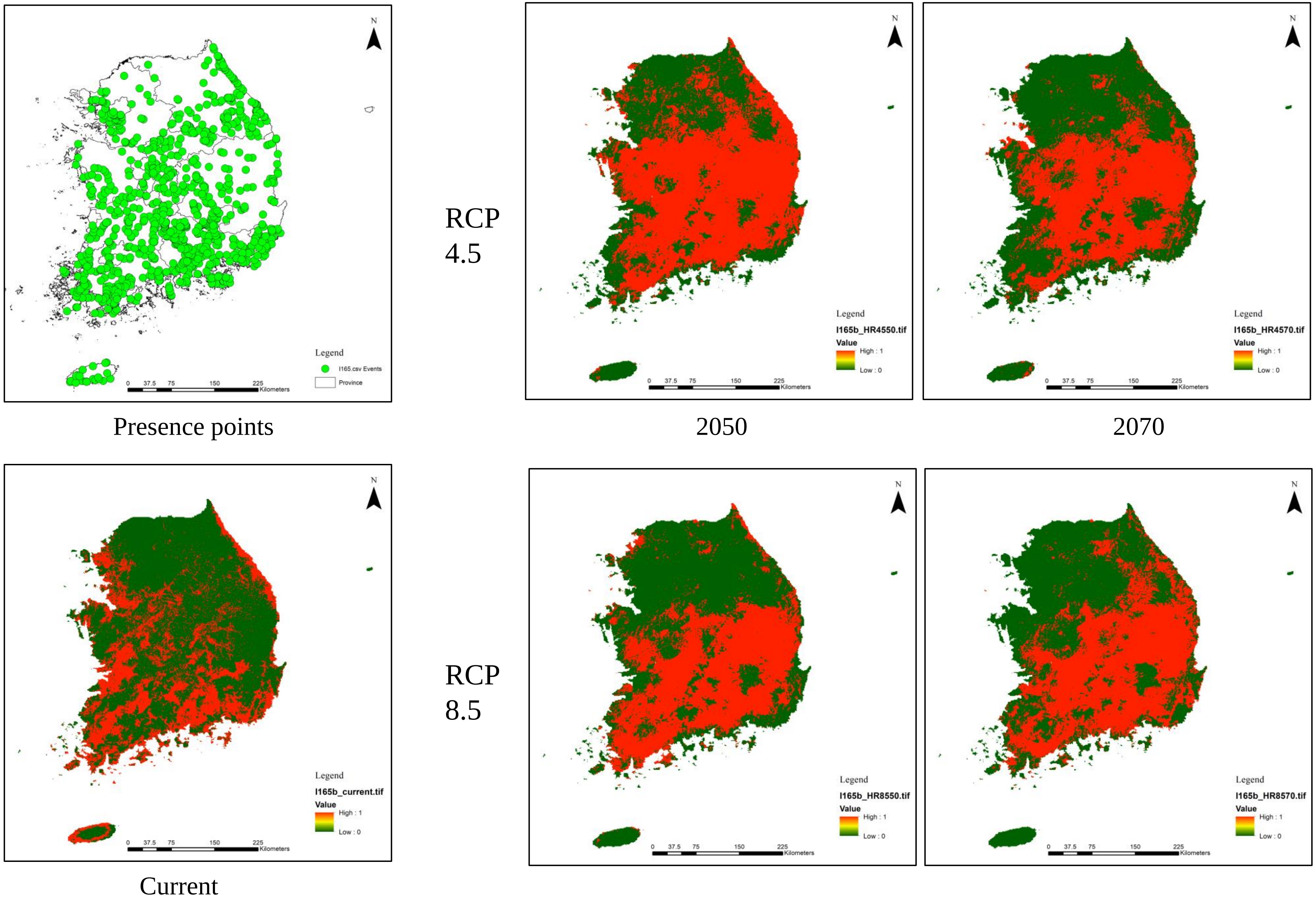

Figure S1f. Spatial distribution of *Festuca arundinacea* (I165) under RCP 4.5 and RCP 8.5.

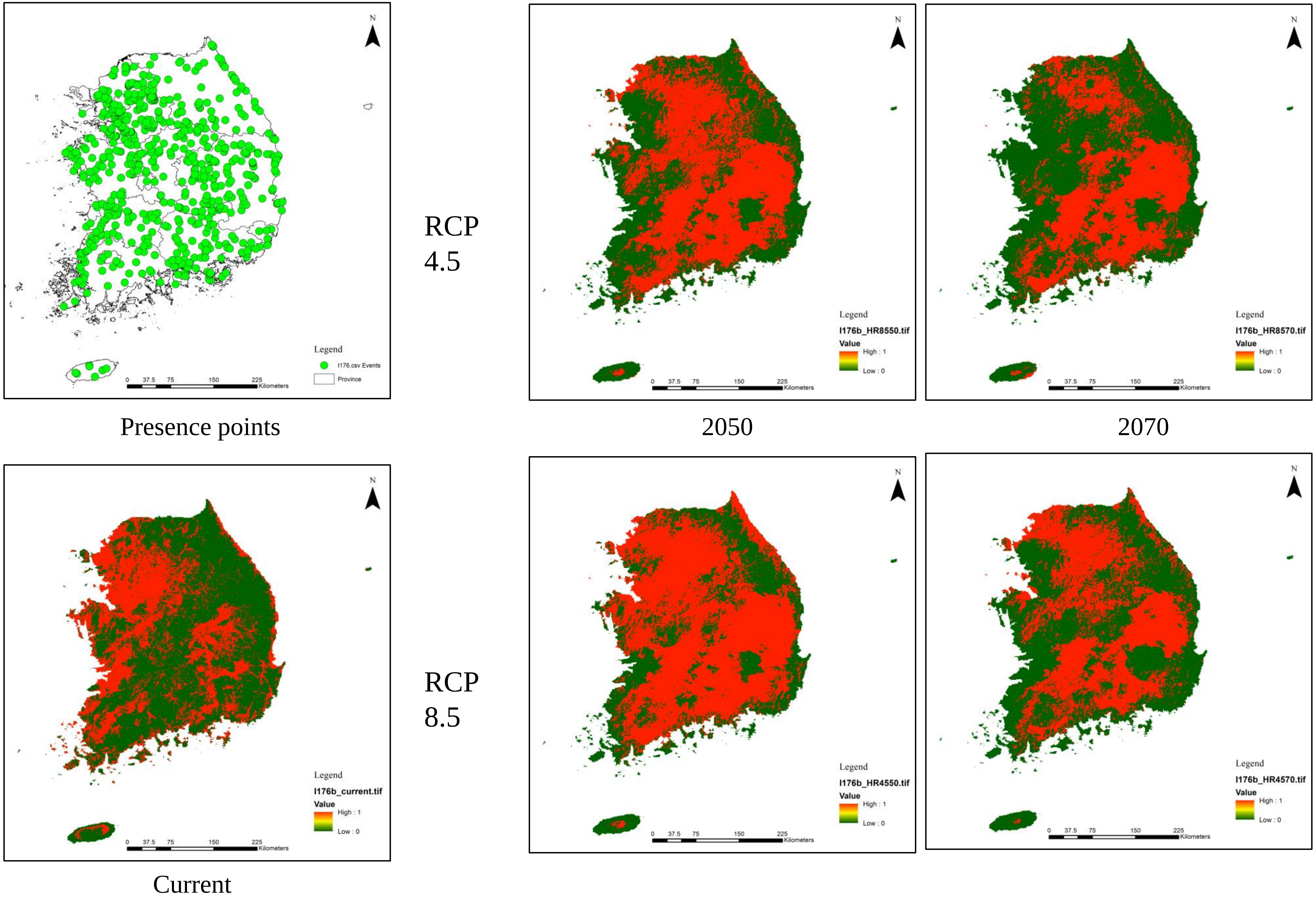

Figure S1g. Spatial distribution of *Helianthus tuberosus* (I176) under RCP 4.5 and RCP 8.5.

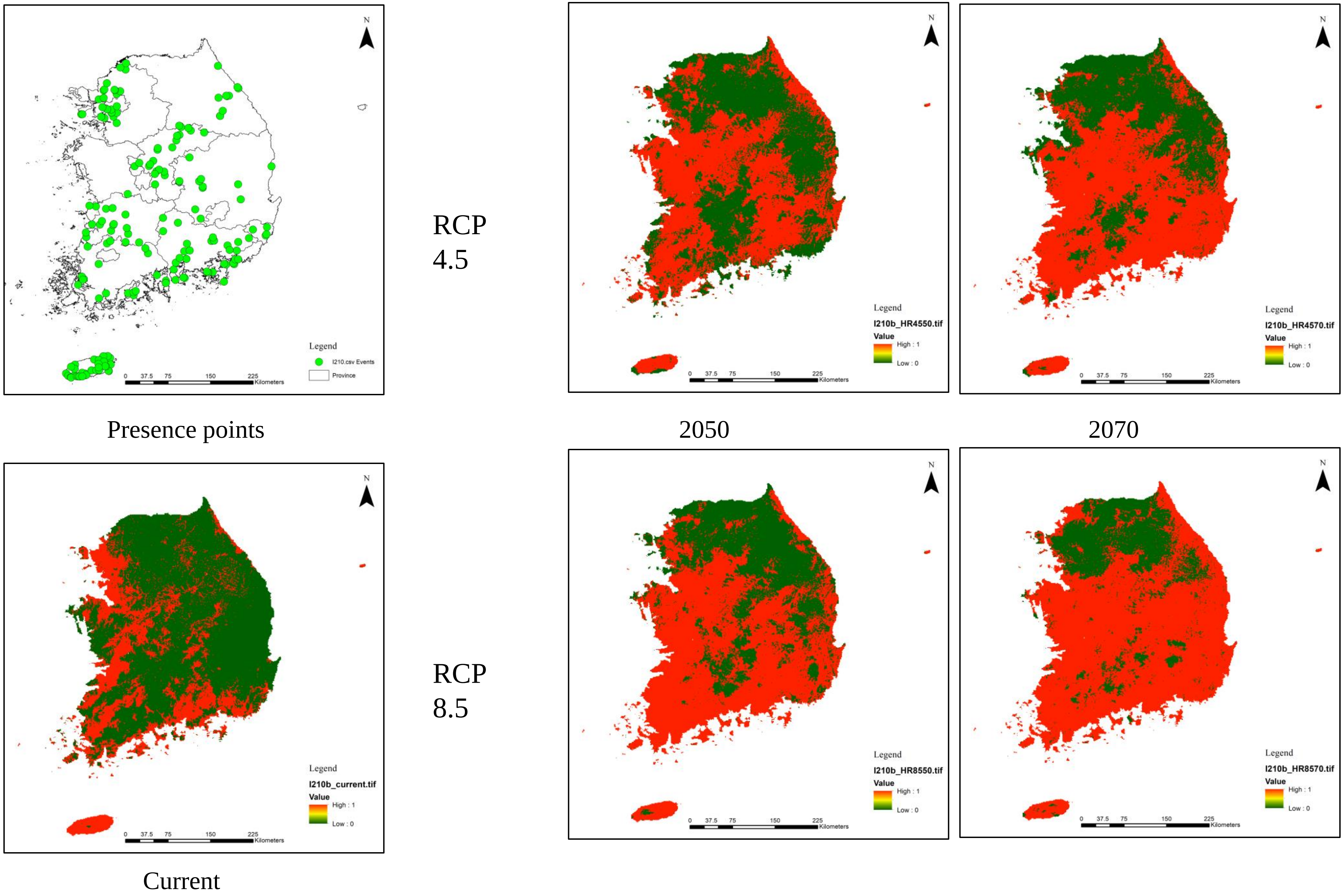

Figure S1h. Spatial distribution of *Lolium perenne* (I210) under RCP 4.5 and RCP 8.5.

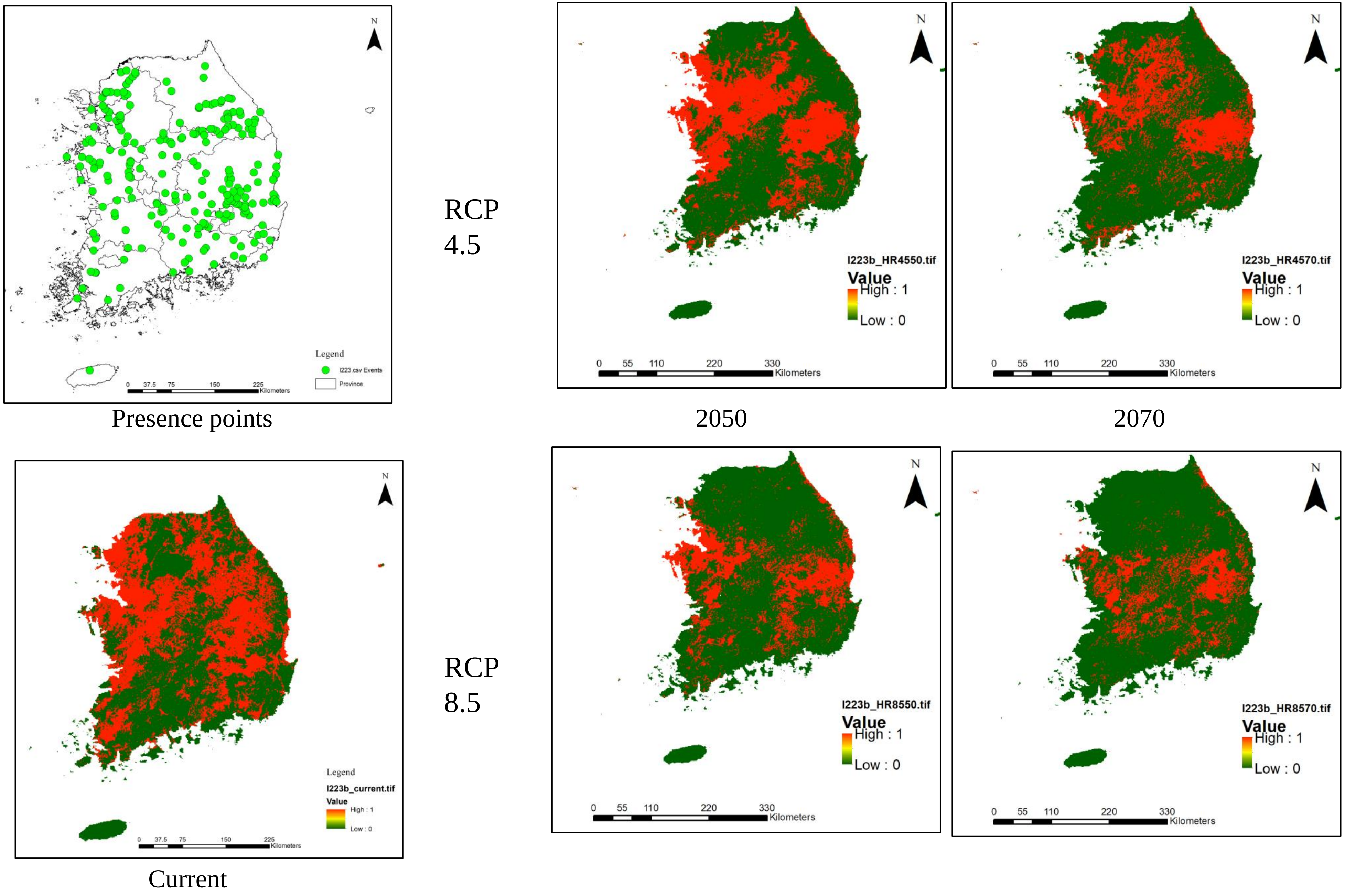

Figure S1i. Potential risk area of *Medicago sativa* (I223) under RCP 4.5 and RCP 8.5.

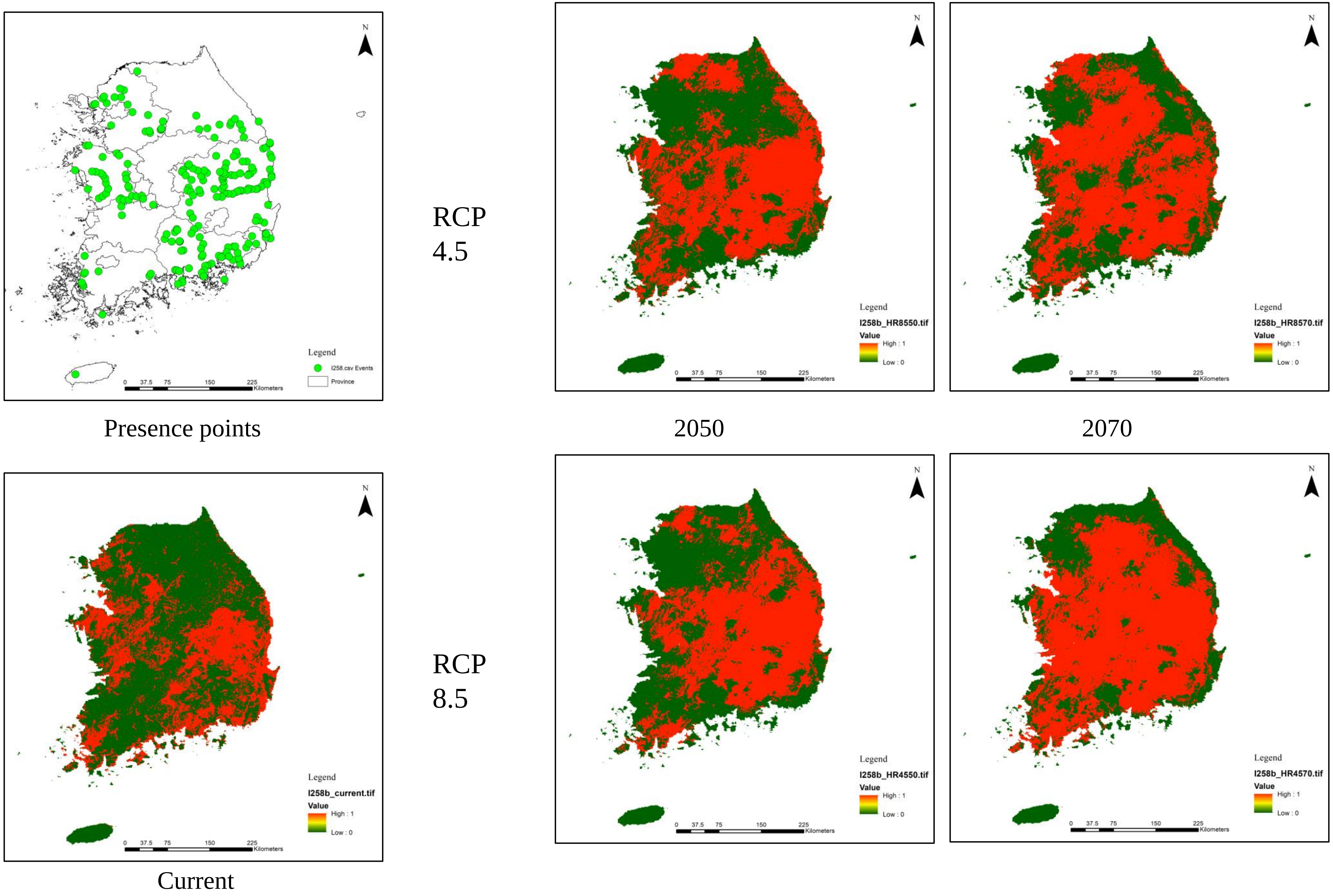

Figure S1j. Spatial distribution of *Poa pratensis* (I258) under RCP 4.5 and RCP 8.5.
