# Supplementary material for: Assessment of the Spatial Invasion Risk of Intentionally Introduced Alien Plant Species (IIAPS) under Environmental Change in South Korea
Source: Biology (Basel). 2021 Nov 12;10(11):1169. doi: 10.3390/biology10111169 (PMC8614709; doi:10.3390/biology10111169)
Supplement: Supplementary file 1 [file biology-10-01169-s001.zip › Figure S2.pdf]

**Figure S2a-j.** The Jackknife test results for indicating the relative contribution of environmental variable for modeling differe nt intentionally introduced alien plant species. **S2**a, *Amorpha fruticosa*; S2b, *Coreopsis lanceolata*; S2c, *Dactylis glomerata*; S2d, *Era grostis curvula*; S2e, *Ageratina altissima* ; S2f, *Festuca arundinacea*; S2g *Helianthus tuberosus*; S2h, *Lolium perenne*; S2i *Medicago sativ a*; S2j, *Poa pratensis*

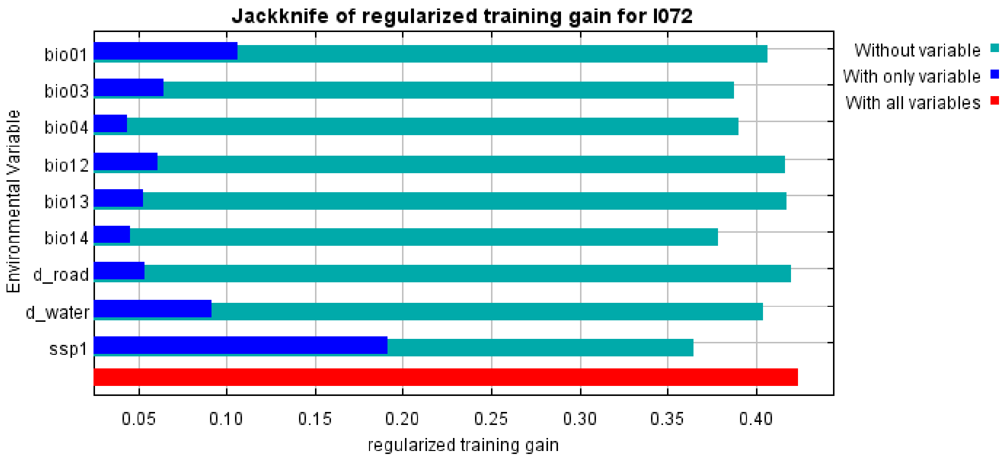


Figure S2a. The Jackknife test results for indicating the relative contribution of environmental varibales for *Amorpha fruticosa*

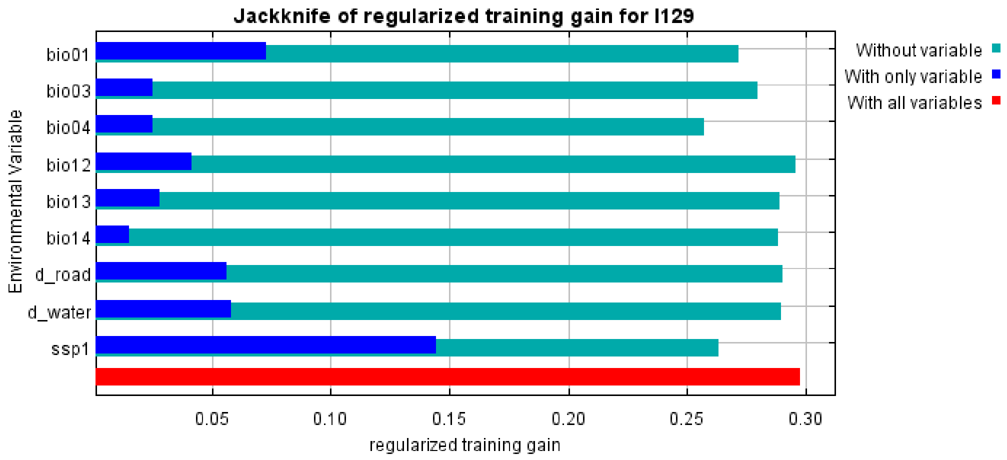


Figure S2b. The Jackknife test results for indicating the relative contribution of environmental varibales for *Coreopsis lanceolata*

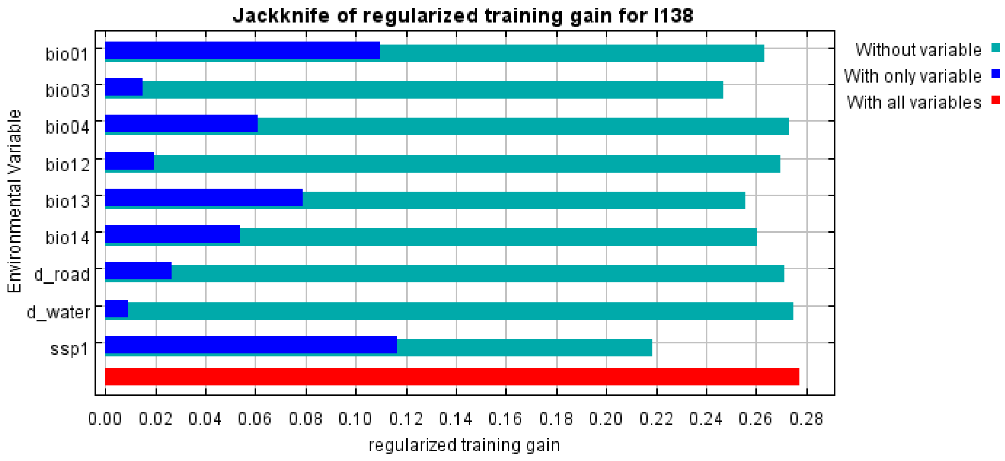


Figure S2c. The Jackknife test results for indicating the relative contribution of environmental varibales for *Dactylis glomerata*

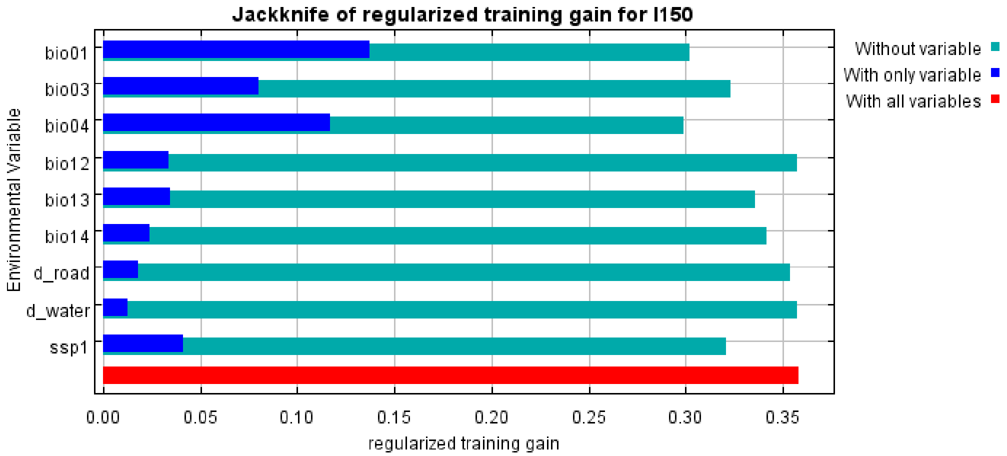


Figure S2d. The Jackknife test results for indicating the relative contribution of environmental varibales for *Eragrostis curvula*

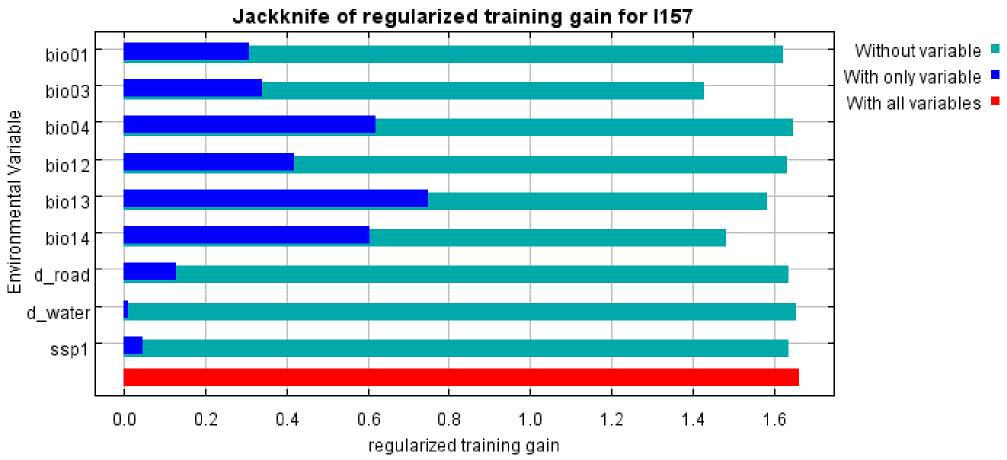


Figure S2e. The Jackknife test results for indicating the relative contribution of environmental varibales for *Ageratina altissima*

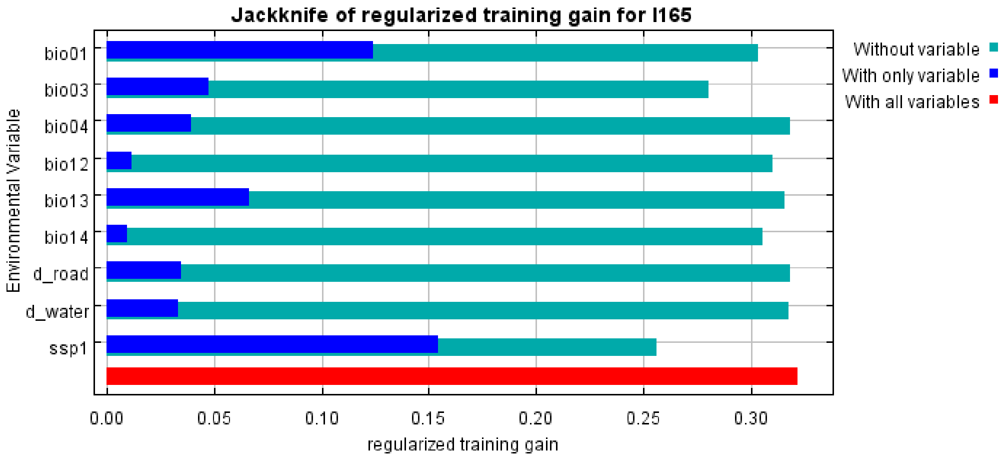


Figure S2f. The Jackknife test results for indicating the relative contribution of environmental varibales for *Festuca arundinacea*

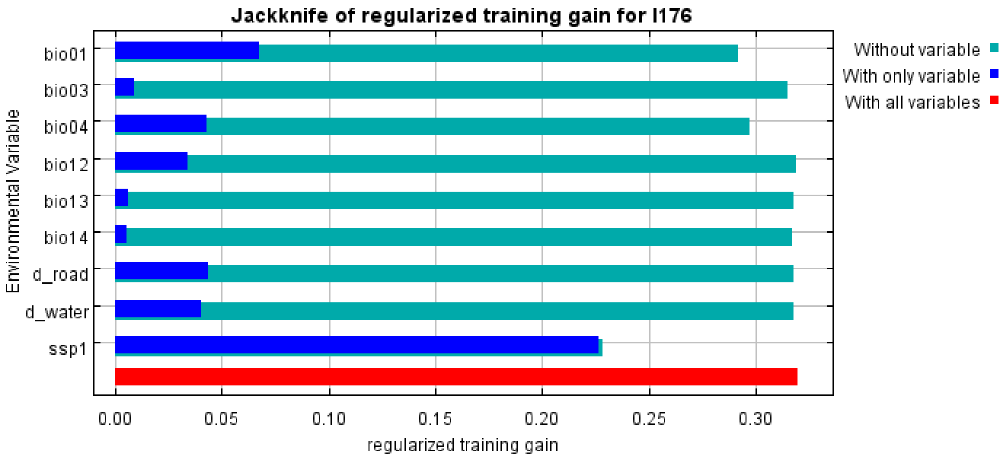


Figure S2g. The Jackknife test results for indicating the relative contribution of environmental varibales for *Helianthus tuberosus*

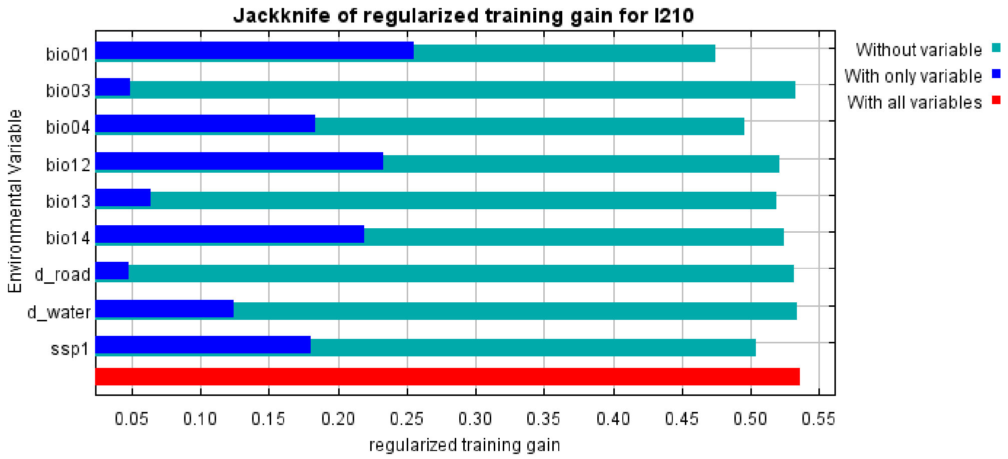


Figure S2h. The Jackknife test results for indicating the relative contribution of environmental varibales for *Lolium perenne*

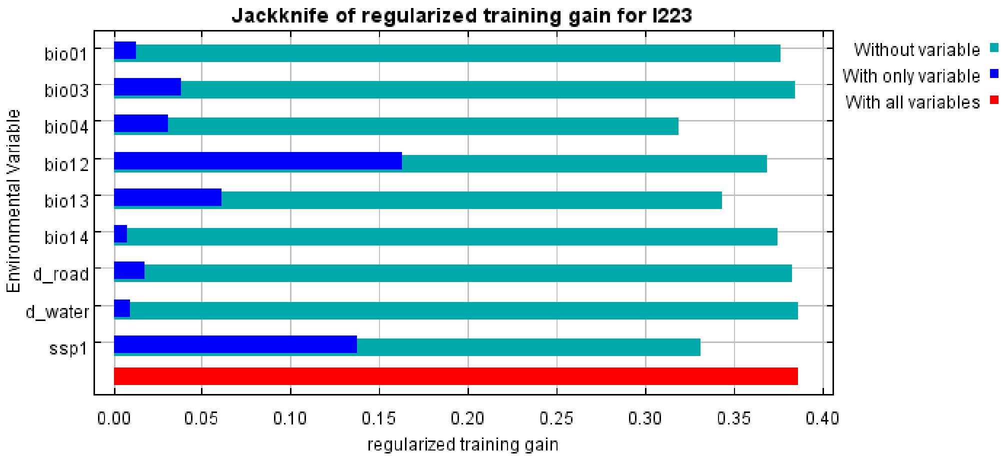


Figure S2i. The Jackknife test results for indicating the relative contribution of environmental varibales for *Medicago sativa*

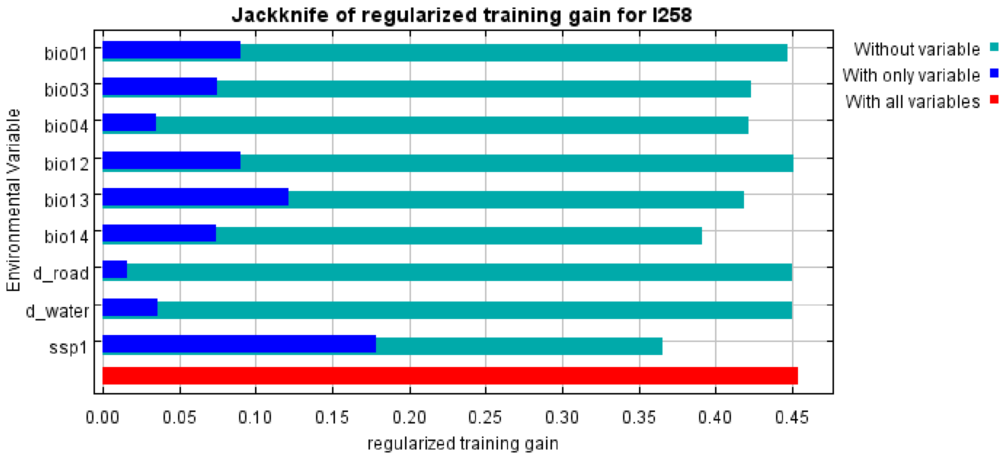


Figure S2j. The Jackknife test results for indicating the relative contribution of environmental varibales for *Poa pratensis*
