# Supplementary material for: Assessment of the Spatial Invasion Risk of Intentionally Introduced Alien Plant Species (IIAPS) under Environmental Change in South Korea
Source: Biology (Basel). 2021 Nov 12;10(11):1169. doi: 10.3390/biology10111169 (PMC8614709; doi:10.3390/biology10111169)
Supplement: Supplementary file 1 [file biology-10-01169-s001.zip › Figure S3.pdf]

Figure S3a-j. The area under the receiver operating characteristics (ROC) curve for determing the model's goodness of fit under the current climate. **S3**a, *Amorpha fruticosa*; S3b, *Coreopsis lanceolata*; S3c, *Dactylis glomerata*; S3d, *Eragrostis curvula*; S3e, *Ageratina altissima* ; S3f, *Festuca arundinacea*; S3g *Helianthus tuberosus*; S3h, *Lolium perenne*; S3i *Medicago sativa*; S3j, *Poa pratensis*

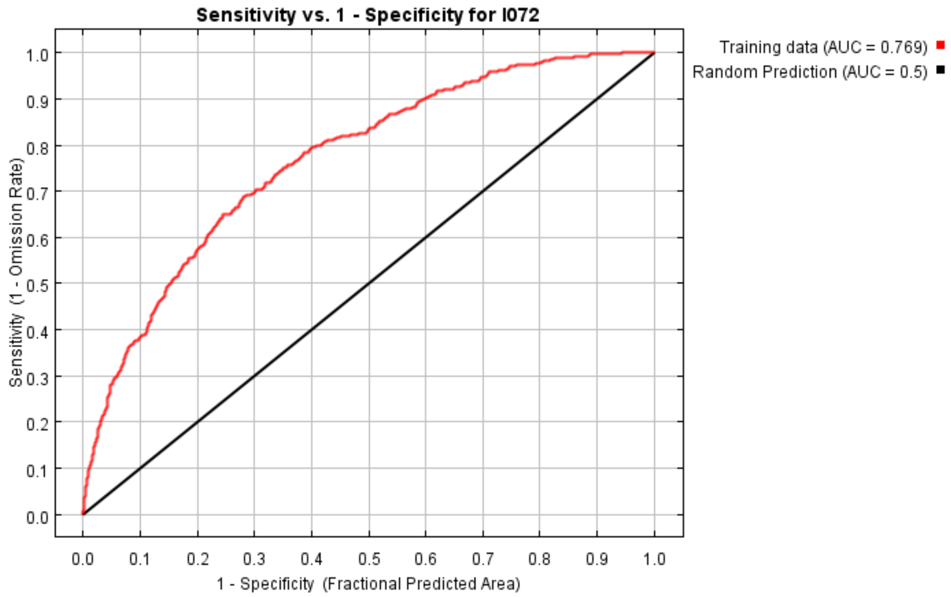

Figure S3a. The ROC curve for *Amorpha fruticosa* under current climate.

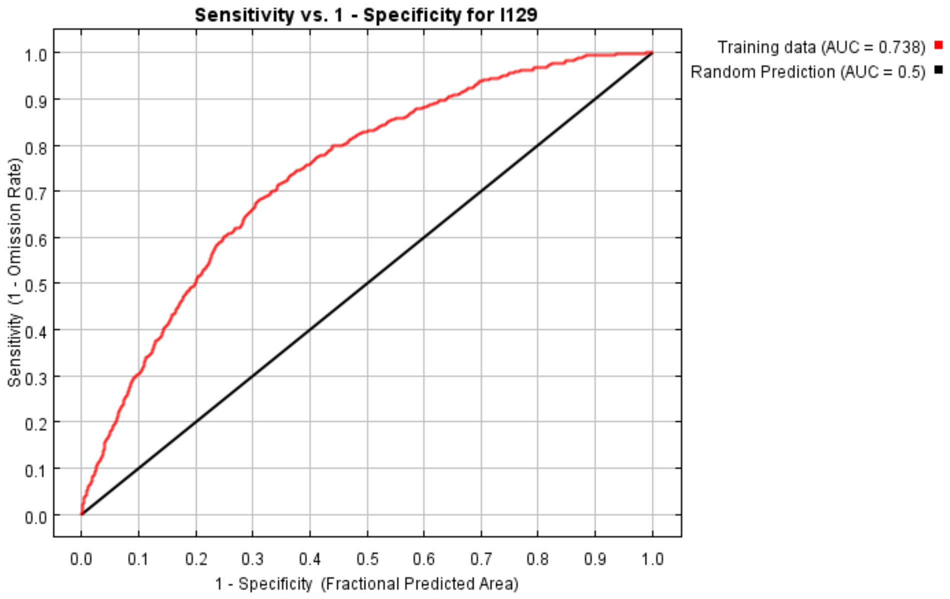

Figure S3b. The ROC curve for *Coreopsis lanceolata* under current climate.

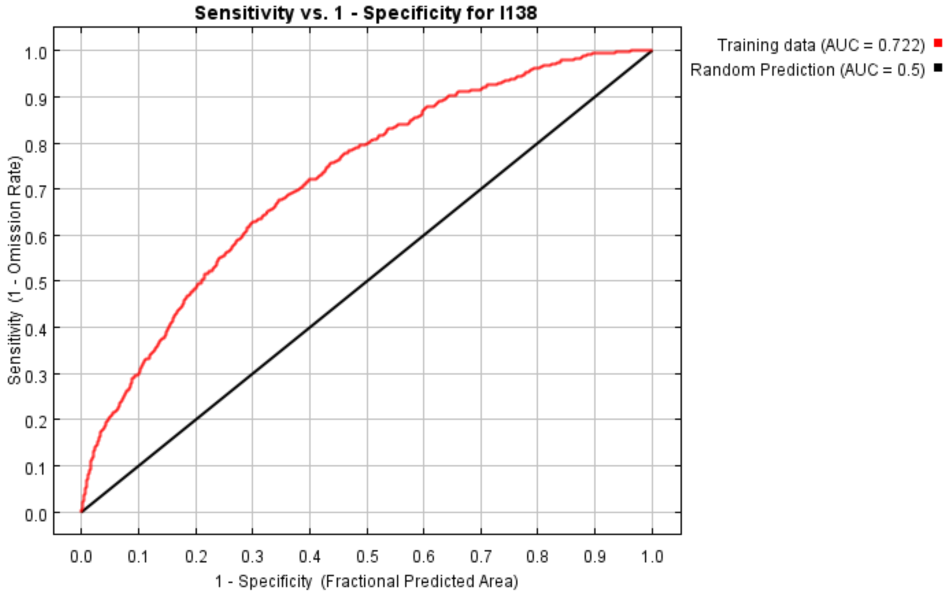

Figure S3c. The ROC curve for *Dactylis glomerata* under current climate.

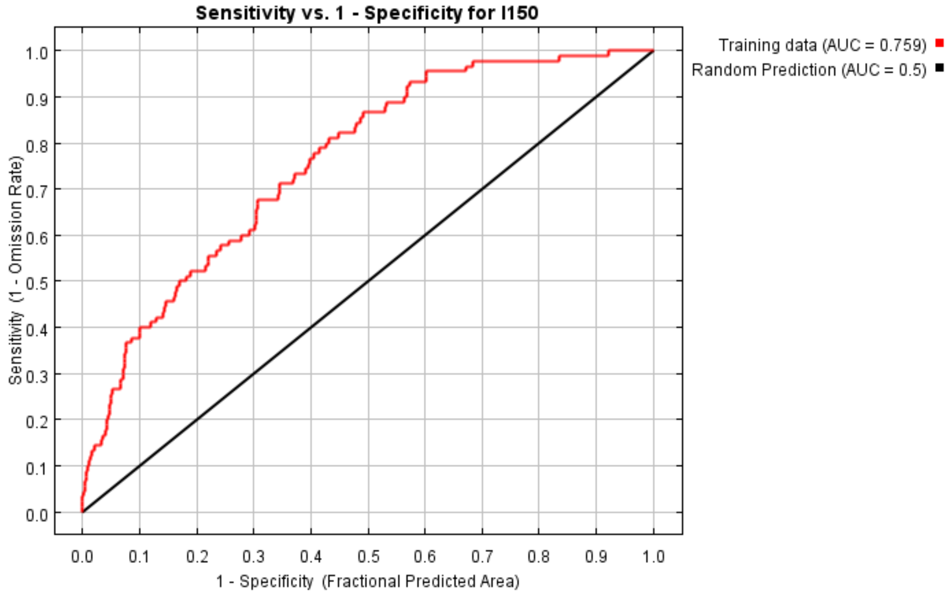

Figure S3d. The ROC curve for *Eragrostis curvula* under current climate.

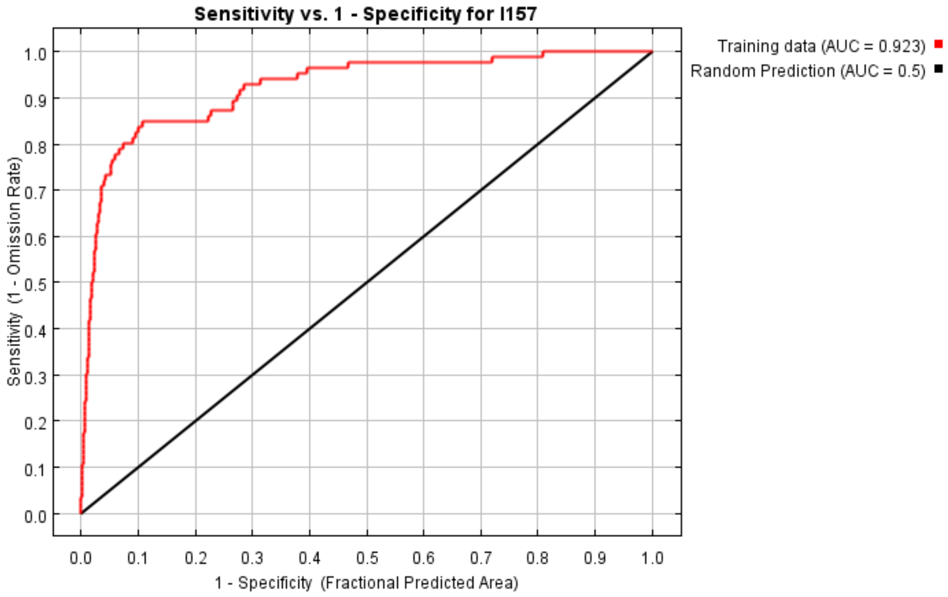

Figure S3e. The ROC curve for *Ageratina altissima* under current climate.

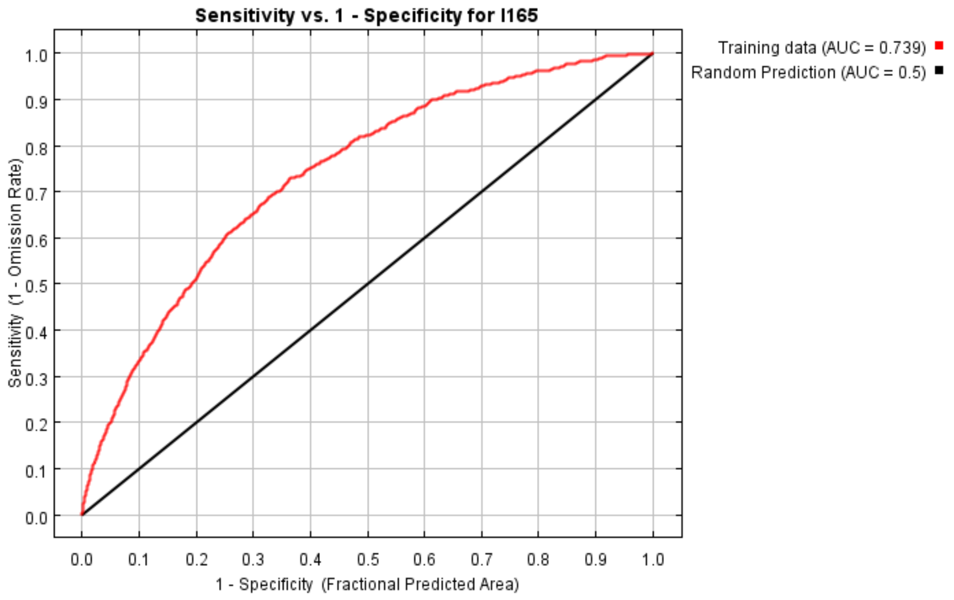

Figure S3f. The ROC curve for *Festuca arundinacea* under current climate.

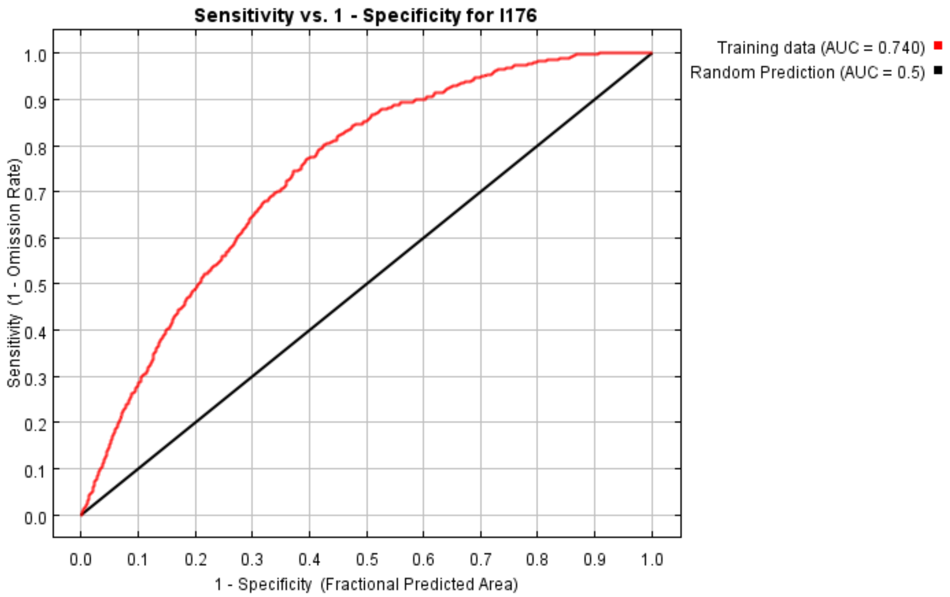

Figure S3g. The ROC curve for *Helianthus tuberosus* under current climate.

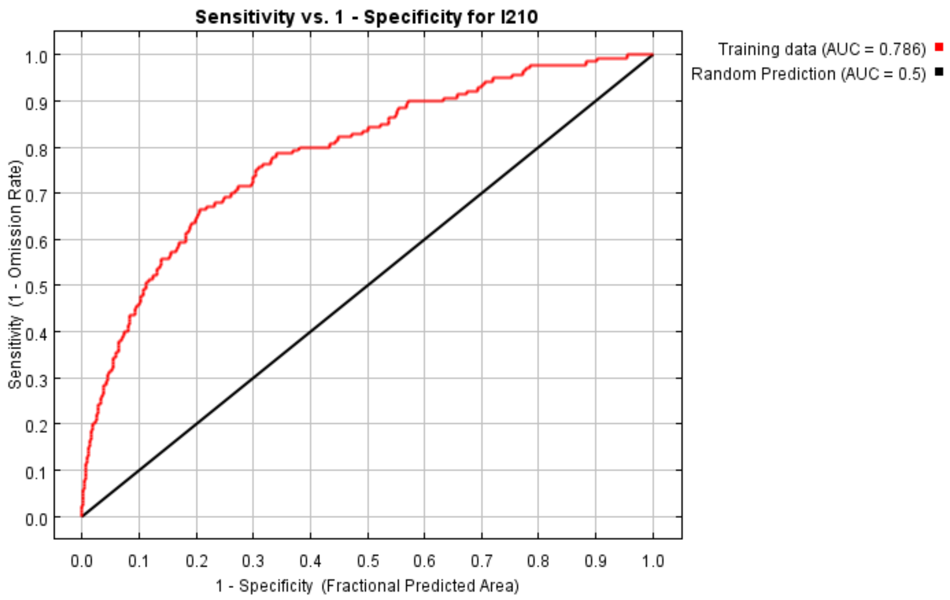

Figure S3h. The ROC curve for *Lolium perenne* under current climate.

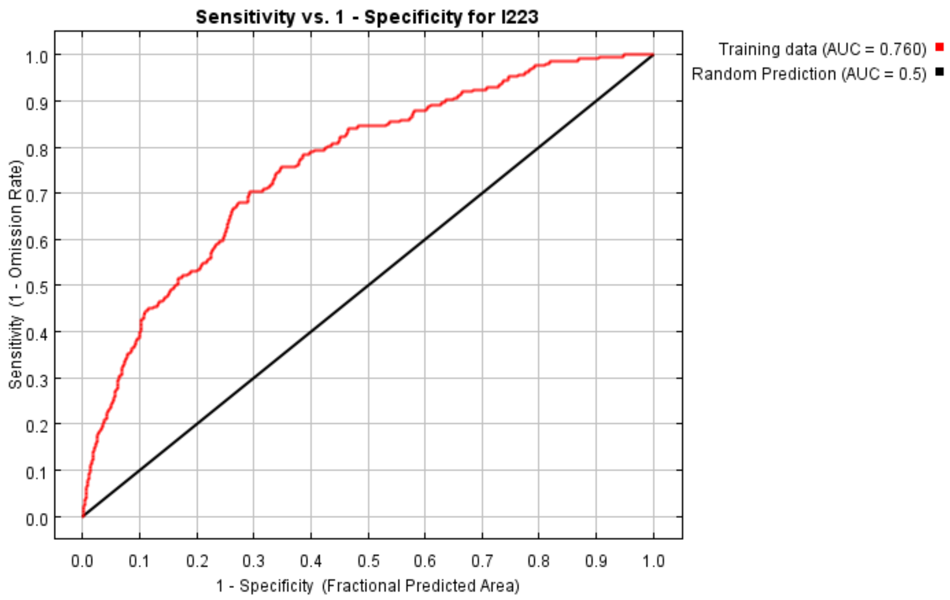

Figure S3i. The ROC curve for *Medicago sativa* under current climate.

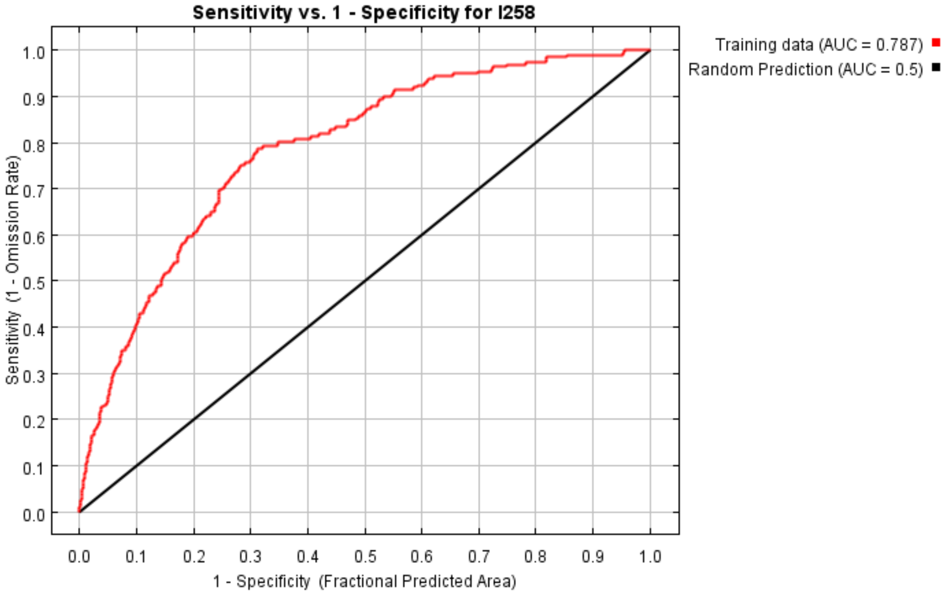

Figure S3j. The ROC curve for *Poa pratensis* under current climate.
