# Supplementary material for: Assessment of the Spatial Invasion Risk of Intentionally Introduced Alien Plant Species (IIAPS) under Environmental Change in South Korea
Source: Biology (Basel). 2021 Nov 12;10(11):1169. doi: 10.3390/biology10111169 (PMC8614709; doi:10.3390/biology10111169)
Supplement: Supplementary file 1 [file biology-10-01169-s001.zip › Figure S4_Average spatial distribution of three groups.pdf]

Figure S4. Comparative analyses of the average spatial distribution of intentionally introduced alien plant species (IIAPS) categorized into three groups under the predicted environmental changes in South Korea.

A

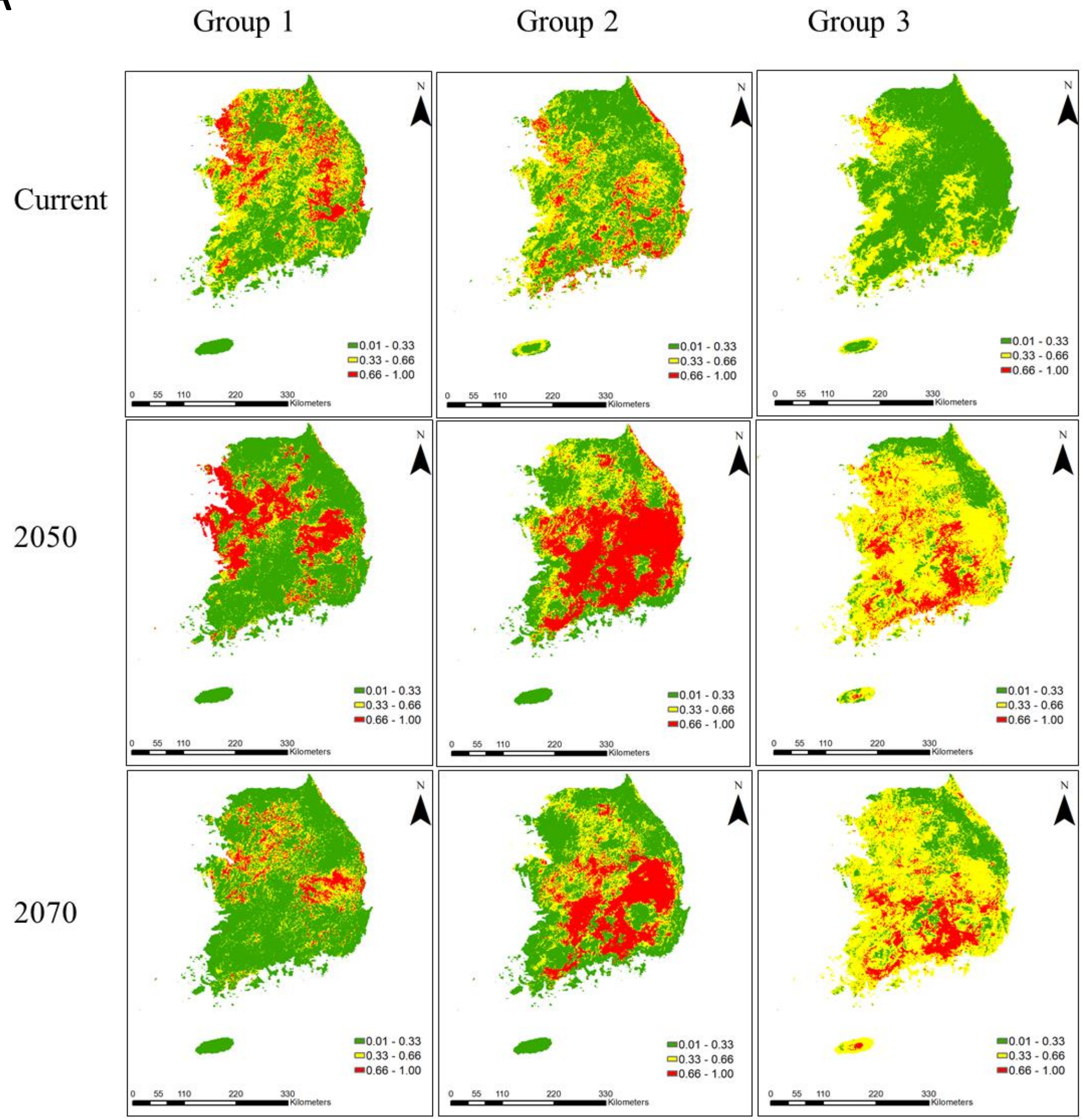
Group 1
Group 2
Group 3
Current
2050
2070
0.01 - 0.33
0.33 - 0.66
0.66 - 1.00
0 55 110 220 330 Kilometers
N

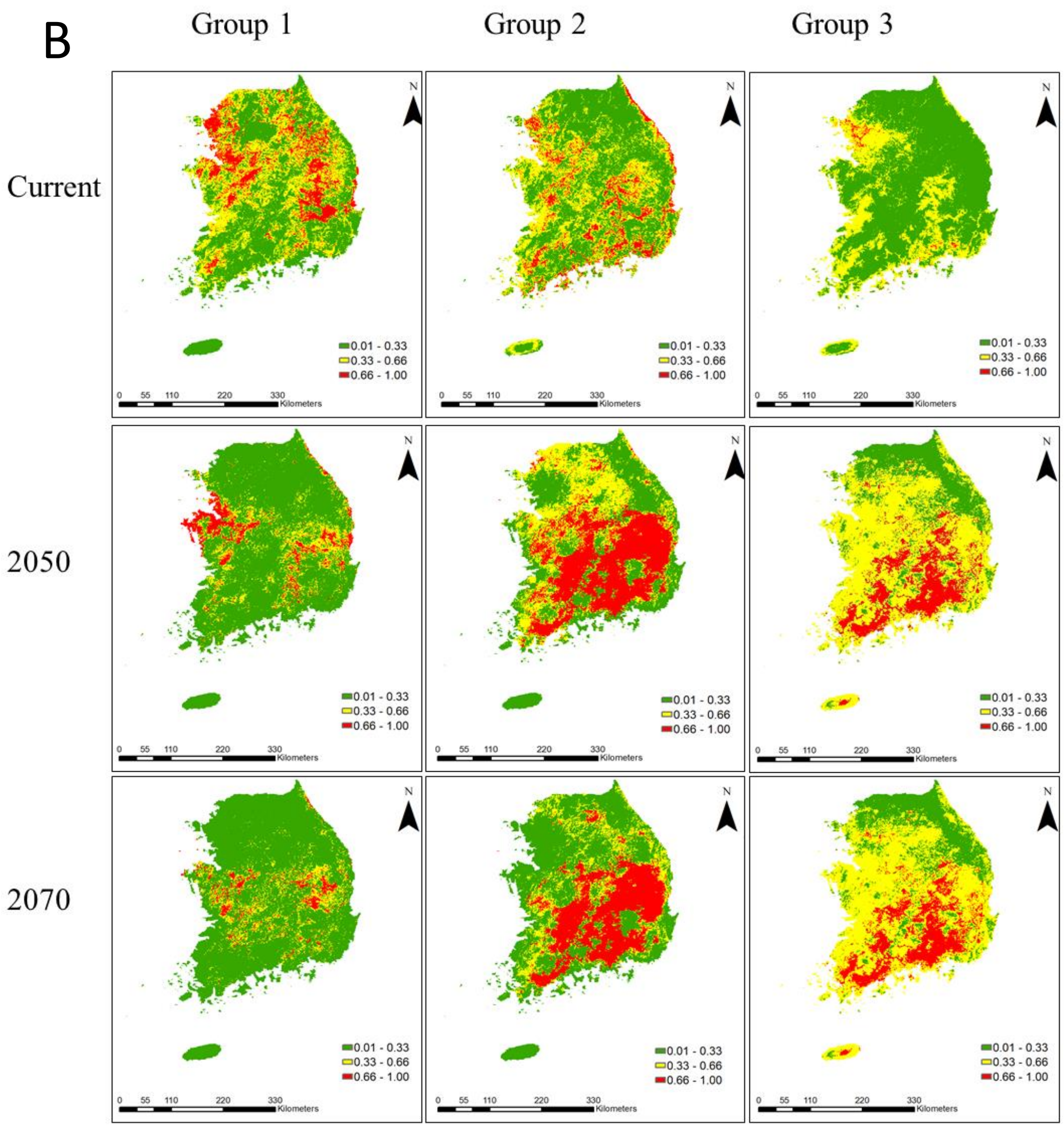

Figure S4. Comparative analyses of the average spatial distribution of intentionally introduced alien plants (IAPs) under the predicted environmental changes in South Korea. IAPs are clustered into three groups: group 1 (n=1), group 2 (n=5), and group 3 (n=4). The details of introduced alien plants (IAPs) corresponding to each group are presented in Table 1. Current and future spatial distribution of IAPS is predicted under the environment change RCP 4.5 (A), and RCP 8.5 (B). In these figures the cell values 0.01-0.33, 0.33-0.66, and 0.66-1.00 indicate low, moderate, and high spatial distribution of IAPS, respectively.
